# *ZNF139*/*circZNF139* promotes cell proliferation, migration and invasion via activation of PI3K/AKT pathway in bladder cancer

**DOI:** 10.18632/aging.103256

**Published:** 2020-05-26

**Authors:** Jie Yao, Kaiyu Qian, Chen Chen, Xiaoping Liu, Donghu Yu, Xin Yan, Tongzu Liu, Sheng Li

**Affiliations:** 1 Department of Biological Repositories, Zhongnan Hospital of Wuhan University, Wuhan 430071, China; 2 Human Genetics Resource Preservation Center of Hubei Province, Wuhan 430071, China; 3 Department of Urology, Zhongnan Hospital of Wuhan University, Wuhan 430071, China

**Keywords:** circular RNA, ZNF139, bladder cancer, PI3K/AKT signaling pathway, bioinformatics

## Abstract

Existing reports identify the involved roles of *ZNF139* and its one circular RNA (circRNA), circZNF139, in the progression of various tumors. However, their relevance and function role in bladder cancer (BC) remain largely unexplored. Herein, we aimed to reconnoiter the role and potential mechanism of *ZNF139* and *circZNF139* in the progression of BC. Firstly, bioinformatics analyses indicated *ZNF139* was upregulated in BC tissues and correlated with disease-free survival of BC patients. The subcellular localization and structural analyses of *ZNF139* conveyed the possibility of *ZNF139* functioning as a transcription factor. Secondly, *circZNF139* was validated by bioinformatics analyses and RNase R tests. *ZNF139* and *circZNF139* were both significantly upregulated in BC cell lines. Functionally, *ZNF139*/*circZNF139* had facilitated effects on the proliferative, clonal, migratory, and invasive potential of BC cells. Mechanistically, GO, KEGG pathway analyses and western blot assays altogether unveiled *ZNF139*/*circZNF139* activated PI3K/AKT pathway in BC cells, supported by the alteration of AKT at phosphorylation level and PI3K at the protein level. Collectively, this work reveals *ZNF139* and *circZNF139* cooperate closely with each other to promote cell proliferation, migration and invasion via activation of PI3K/AKT pathway in BC.

## INTRODUCTION

Ranked as the tenth most repeatedly diagnosed tumor in the global, bladder cancer (BC) is a heterogeneous group of tumors with various histological subgroups [1] and entails the highest cost for each patient among cancers of all stripes [2]. Most BCs are urothelial carcinomas, previously known as transitional cell carcinomas, which is a cancer sourced from the cells in the inner lining of the bladder. In term of the histopathological characteristics, urothelial carcinomas can be commonly subdivided into conventional urothelial carcinoma, urothelial carcinoma with diverging differentiation or variant urothelial carcinoma [3]. The gold standard for treatment is a radical cystectomy preceded, in combination with chemotherapy or bladder instillations of BCG or mitomycin when possible to prevent the distant spread [4]. Despite the advance in these invasive treatments, BCs still have recurrences with high rates and patients suffered have associated worse outcome. Currently, cystoscopy is the gold standard for BC diagnosis and surveillance. Unfortunately, its high frequency not only brings great pains to patients and lowers their life quality, but also causes high financial burden to individuals and nations [5]. Accordingly, it is still urgently needed to inspect the molecular pathogenesis of this disease and identify effective biomarkers.

Sourced from precursor mRNA, circular RNA (circRNA) was first identified in the early 1990s and represents a large number of non-coding RNAs [6]. After that, many circRNAs highly abundant and conserved among species have continuously been reported [7–10]. They are covalently closed with circular structure, have tissue-specific and cell-specific expression patterns, and are regulated by specific cis-acting elements and trans-acting factors [11]. Despite these valuable advances in understanding circRNAs, their functions remain largely unexplored. Numerous circRNAs are reported to exert vital biological effects by acting as microRNA or protein sponges, such as well-studied *ciRS-7* and *circHIPK3* [12, 13]. Additionally, other circRNAs can interact with RNA-binding proteins or be translated into proteins, as respectively exemplified by *circMb1* [14] and *circZNF609* [15]. Recent developments in the field of circRNAs have led to an increasing interest in characterizing their relevance in bountiful diseases. Specially, many studies focused on the role of circRNAs in tumor development and their biomarker potential reveal that circRNAs correlated with clinical pathological characteristics represent an attractive new class of biomarkers, such as for diagnosis, prognosis and prediction [16–19]. In spite of the emerging BC-associated circRNAs, such as *circHiPK3* [20] and *circBCRC-3* [21], other circRNAs’ relevance and function in BC have not been thoroughly addressed and remain largely unclear.

As one member of the zinc finger protein family, zinc finger with KRAB and SCAN domains 1 (*ZNF13*9, also termed *ZKSCAN1*) plays regulatory roles in the transcriptional level of multifold genes and is related to the development and progression of tumors. Convincing results have appeared from studies of gastric cancer [22, 23] and hepatocellular carcinoma [24]. However, very little is currently known about the relevance and function of *ZNF139* in BC. According to Jeck et al. [25] and Salzman et al. [26], a covalently linked 668-nt circRNA termed *circZNF139* is generated by splicing exons 2 and 3 of *ZNF139* together. This circRNA is particularly abundant in human brain and liver [27], and has been reported to be implicated in the development of hepatocellular carcinoma [28]. However, its function role in BC remains largely unknown.

In this work, we aimed to investigate the role and preliminary potential mechanism of *ZNF139* and its circRNA (*circZNF139*) in regulating the progression of BC. First, the expression of *ZNF139* in BC tissues, its correlation with the BC prognosis, as well as its localization and structure were assessed according to bioinformatics analyses. Second, co-expression genes related with *ZNF139* in BC were evaluated by Gene Ontology (GO) and Kyoto Encyclopedia of Genes and Genomes (KEGG) pathway analyses. The *circZNF139* was validated, and the effect of *ZNF139*/*circZNF139* on the proliferative, clonal, migratory, and invasive potential of BC cells was investigated, respectively. Finally, the association of *ZNF139/circZNF139* with the PI3K/AKT signaling pathway in BC was primarily explored.

## RESULTS

### Bioinformatics analyses of ZNF139

**Figure 1:**
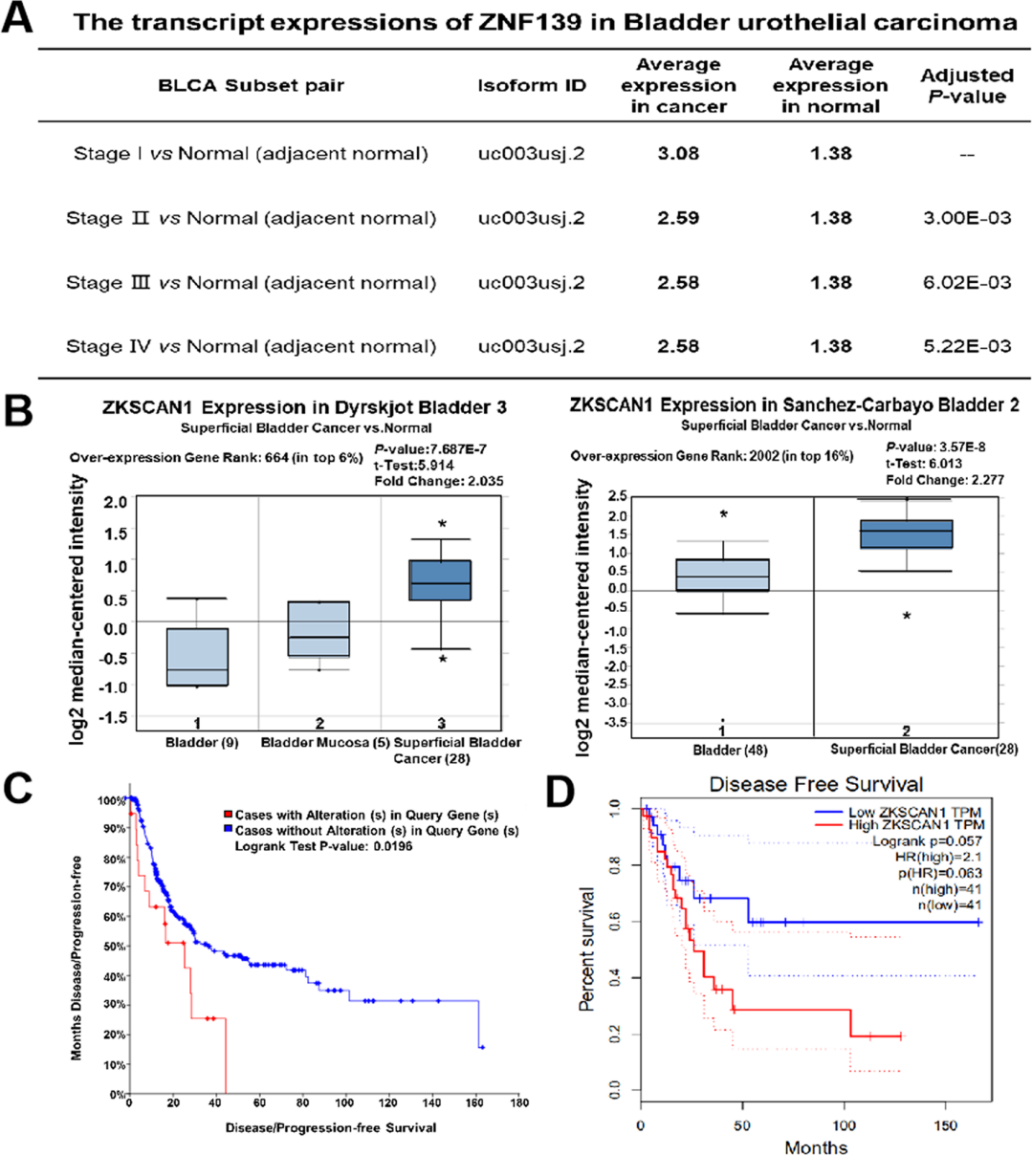
**Upregulation of *ZNF139* in BC tissues and its correlation with the prognosis of BC patients.** (**A**) The transcript expressions of *ZNF139* were significantly higher in bladder urothelial carcinoma at varied stages (Stage I, II, III, IV) than in adjacent normal as analyzed in CRN database (http://syslab4.nchu.edu.tw). (**B**) Box plots showed expression of *ZKSCAN1* (namely *ZNF139*) at mRNA level in Dyrskjot Bladder 3 (left panel) and Sanchez-Carbayo Bladder 2 (right panel), respectively. The shown indicators include over-expression gene rank, associated *P* values, statistical analysis and fold change according to Oncomine (http://www.oncomine.org). The association of *ZNF139* expression with the disease-free survival was analyzed by (**C**) cBioPortal database (http://cbioportal.org) and (**D**) GEPIA database (http://gepia.cancer-pku.cn/). ZNF139, zinc finger with KRAB and SCAN domains 1; BC, bladder cancer; CRN, CancerRNA-Seq Nexus.

Initially, the transcription levels of *ZNF139* in multifarious studies were evaluated from Cancer RNA-Seq Nexus (CRN, http://syslab4.nchu.edu.tw) and Oncomine (http://www.oncomine.org) databases. According to Data in CRN database, the transcript expressions of ZNF139 were significantly higher in bladder urothelial carcinoma at Stage I/II/III/IV than in adjacent normal (Figure 1A). As presented in Figure 1B, further analysis of multiple BC samples in Oncomine consistently showed higher expression of *ZNF139* in superficial BC than normal. It thus followed that *ZNF139* is upregulated in BC tissues. Next, the association of *ZNF139* expression with the prognosis of BC patients was analyzed by cBioPortal database (http://cbioportal.org) and GEPIA database (http://gepia.cancer-pku.cn/). The results demonstrated that cases with alteration in query gene (namely *ZNF139*) had evidently worse disease-free survival than cases without alteration in *ZNF139* (Figure 1C) and high expression of ZNF139 was associated with worse disease free survival (Figure 1D). Taken together, all results presented above indicated that *ZNF139* is upregulated in BC tissues and correlated with the disease-free survival of BC patients according to bioinformatics analyses.

**Figure 2:**
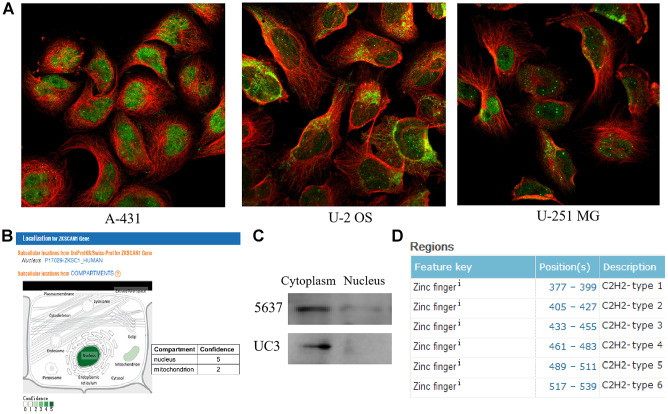
**The subcellular localization and structural analyses of *ZNF139*.** (**A**) The subcellular localization for *ZNF139* obtained from Human Protein Atlas database (https://www.proteinatlas.org/), green represents antibody and red represents microtubules. (**B**) The subcellular localization for *ZNF139* obtained from GeneCards database (https://www.genecards.org/). (**C**) The subcellular localization for *ZNF139* in BC cells as analyzed by western blot assays. (**D**) The structural analysis of *ZNF139* based on UniProt database (https://www.uniprot.org/). BC, bladder cancer.

The Human Protein Atlas database (https://www.proteinatlas.org/) and GeneCards database (https://www.genecards.org/) were then employed to analyze the subcellular localization of *ZNF139*. As clearly shown in Figure 2A, *ZNF139* was mainly localized to the nuclear bodies and its additional location was nucleoplasm and mitochondria in human epidermal cancer A-431 cells, human osteosarcoma U-2 OS cells, and human glioma U-251 MG cells. The result from GeneCards demonstrated two compartments, nucleus with confidence of 5 and mitochondrion with confidence of 2 (Figure 2B), indicating that *ZNF139* was principally located in the nucleus. We then isolated nuclear and cytoplasmic proteins in BC cells, and detected the expression of ZNF139 in them by western blot assays. The results showed that in BC cells, ZNF139 was expressed in both the cytoplasm and the nucleus, and the expression in the cytoplasm was significantly higher than that in the nucleus (Figure 2C). As a member of the zinc finger protein family, ZNF139 protein contained C_2_H_2_- structure specific to transcription factors according to UniProt (https://www.uniprot.org/) as displayed in Figure 2D. Combined with the above-mentioned results, they pointed to the conjecture that *ZNF139* may function as a transcription factor.

### GO and KEGG pathway analyses of co-expression genes related with *ZNF139*

**Figure 3:**
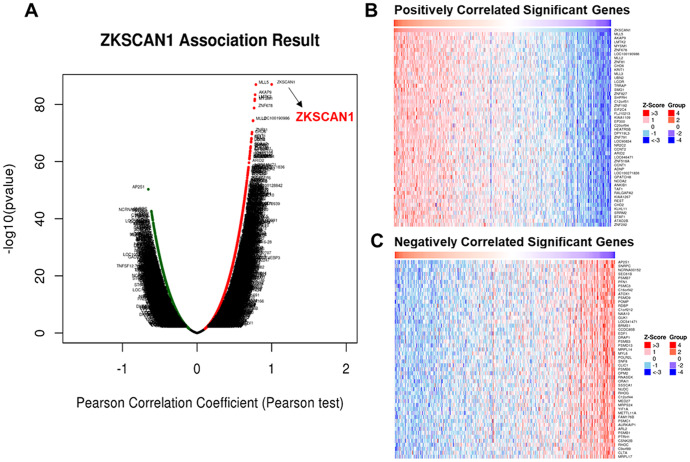
**Genes differentially expressed in association with *ZNF139* in BC according to LinkedOmics.** (**A**) The correlation between *ZKSCAN1* (*i.e.ZNF139*) and genes differentially expressed in BC (413 samples) was evaluated by Pearson test. (**B**) Heat maps showed the top-50 significant genes positively and (**C**) negatively correlated with *ZKSCAN1* (*i.e.ZNF139*) in BC. The red stands for positively correlated genes and the blue stands for negatively correlated genes. ZNF139/ZKSCAN1, zinc finger with KRAB and SCAN domains 1; BC, bladder cancer.

The mRNA sequencing data from 408 BC patients in the TCGA were analyzed by *LinkFinder* module of LinkedOmics (http://www.linkedomics.org/login.php) database. The volcano plot displayed 7,077 genes (dark red plots) were significantly positively correlated with *ZNF139*, while 5,942 genes (dark green plots) were significantly negatively correlated with *ZNF139* (*P*-value<0.05), as clearly shown in Figure 3A. In addition, the top-50 significant genes positively and negatively correlated with *ZNF139* were separately shown in the heat maps (Figure 3B, 3C), and also listed in Supplementary Tables 1 and 2.

**Figure 4:**
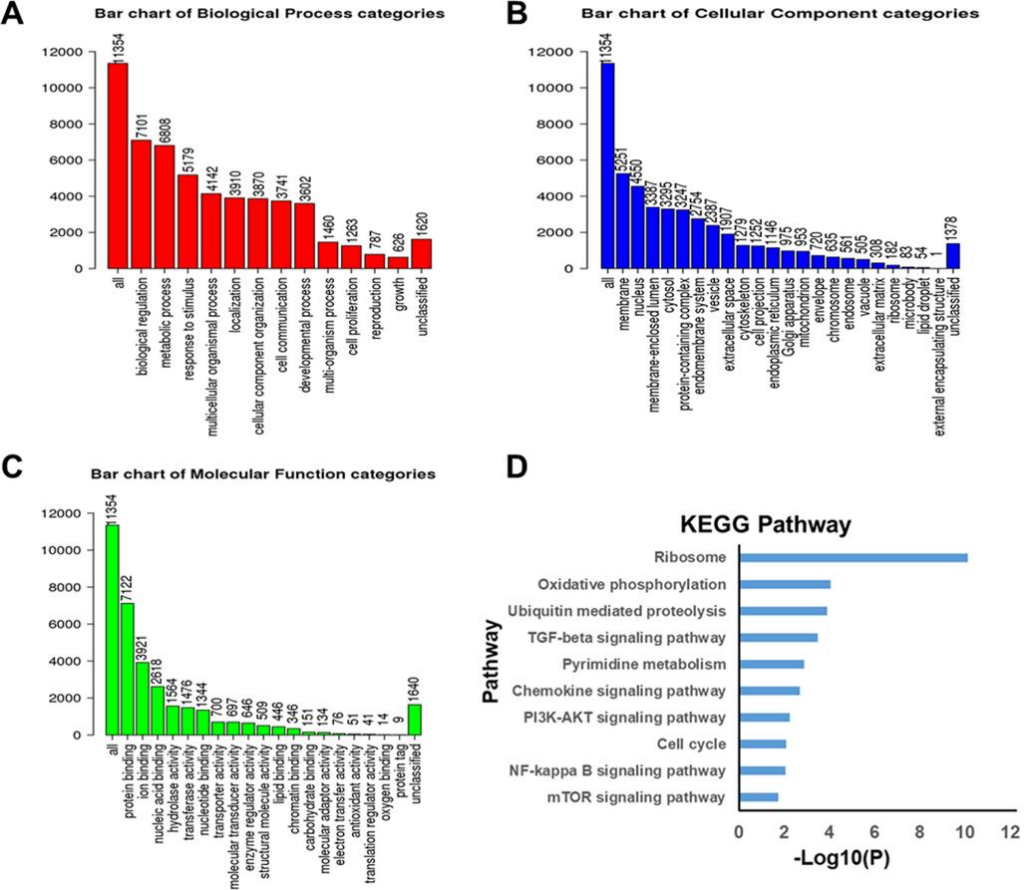
**Significantly enriched GO annotations and KEGG pathways of genes differentially expressed in correlation with *ZNF139* in BC as analyzed using GSEA.** (**A**) Biological processes. (**B**) Cellular component. (**C**) Molecular Function. (**D**) KEGG pathway analysis. GO, Gene Ontology; KEGG, Kyoto Encyclopedia of Genes and Genomes (KEGG); ZNF139, zinc finger with KRAB and SCAN domains 1; BC, bladder cancer; GSEA, Gene Set Enrichment Analysis.

GO functional enrichment analysis is commonly involved in biological process, cellular component and molecular function. Here, GO functional enrichment of differentially expressed genes correlated with *ZNF139* in BC was analyzed using Gene Set Enrichment Analysis (GSEA) with the help of the *LinkFinder* module of LinkedOmics. Results revealed that their biological processes were essentially enriched in biological regulation, metabolic process, response to stimulus and others (Figure 4A). With respect to cellular component, they were predominantly concentrated in membrane, nucleus, macromolecular complex and so forth (Figure 4B). As for molecular function, they were generally centralized on protein binding, ion binding, nucleic acid binding and the like (Figure 4C). Subsequently, KEGG analysis conducted as the same approach clearly shown in Figure 4D demonstrated that the significant pathways associated with these genes chiefly belonged to ribosome, oxidative phosphorylation and PI3K/AKT signaling pathway. Collectively, these findings suggested that *ZNF139* may function as a transcription factor to affect related signaling pathways.

### The *circZNF139*, a circRNA form of *ZNF139*, was validated

**Figure 5:**
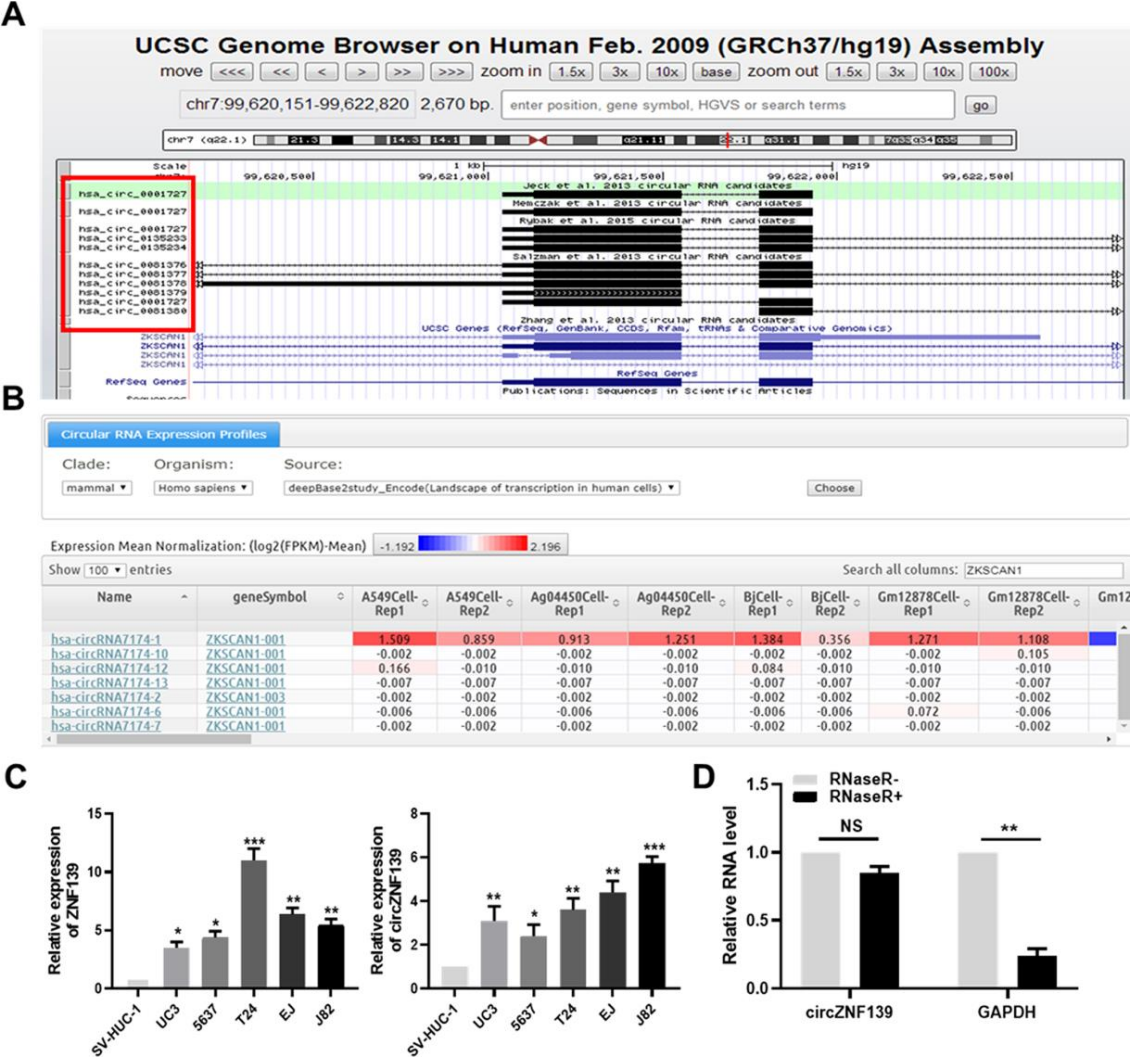
**The validation of *circZNF139*.** (**A**) The circRNA-formed ability of *ZNF139* evaluated by UCSC (http://genome.ucsc.edu/index.html) database. (**B**) Several circRNAs formed by *ZNF139* were evidently differentially expressed in various tumor cells according to DeepBase (https://omictools.com/deepbase-tool) database. (**C**) *ZNF139* (left panel) and *circZNF139* (right panel) were both evidently upregulated in five BC cell lines (UC3, 5637, T24, EJ and J82) compared to one normal cell line SV-HUC-1 detected by qRT-PCR assay. (**D**) The RNA level of predicted circRNA and its control gene GAPDH were evaluated with or without RNase R treatment by qRT-PCR assay. The predicted circRNA, *circZNF139*, was resistant to RNase R treatment. *, *P*<0.05, **, *P*<0.01, ***, *P*<0.001.circ, circular; ZNF139, zinc finger with KRAB and SCAN domains 1; UCSC, University of California, Santa Cruz; BC, bladder cancer; qRT-PCR, quantitative real-time polymerase chain reaction; GAPDH, glyceraldehyde 3-phosphate dehydrogenase.

*ZNF139* has protein-coding ability and may function as a transcription factor on the basis of aforementioned results. Next, we analyzed *ZNF139* in the University of California, Santa Cruz (UCSC, http://genome.ucsc.edu/index.html) database and found some stable circRNAs formed from transcripts of ZNF139 gene (Figure 5A). Confirming to DeepBase (https://omictools.com/deepbase-tool) database, several circRNAs formed by *ZNF139* were evidently differentially expressed in various tumor cells (Figure 5B). Additionally, the expression of circRNAs in various samples was summarized and presented in Supplementary Figure 1 in accordance with the search of *ZNF139* in circBase (http://www.circbase.org/) database. Clearly, *circZNF139* (circRNA ID: hsa_circ_0001727) with 668 nt spliced length had the widest expression spectrum, which was thus selected for the following work.

The expression of *ZNF139* and its circRNA (*circZNF139*) was separately detected by qRT-PCR assay in five BC cell lines (UC3, 5637, T24, EJ and J82) and one normal cell line SV-HUC-1. What stands out in Figure 5C is that *ZNF139* was significantly upregulated in five BC cell lines (UC3, 5637, T24, EJ and J82) in comparison with normal cell line SV-HUC-1 (*P*<0.05, *P*<0.01, *P*<0.001). So was the case of *circZNF139* expressed in BC cells (Figure 5C, right panel, *P*<0.05, *P*<0.01, *P*<0.001). As a highly processive and hydrolytic 30 to 50 exoribonuclease that does not act on circRNA [29], the enzyme RNase R was used to further confirm the circular characteristics of *circZNF139*. As demonstrated in Figure 5D, level of the control gene GAPDH was obviously altered after RNase R^+^ treatment (*P*<0.01). As expected, level of *circZNF139* showed no obvious change in RNase R^+^ group compared to RNase R^-^ group, revealing *circZNF139* was resistant to RNase R treatment. This also supported that apart from the *ZNF139* mRNA, a natural circRNA form of the *ZNF139* gene existed inside.

### *ZNF139*/*circZNF139* promotes the proliferative, clonal, migratory, and invasive potential of BC cells *in vitro*

**Figure 6:**
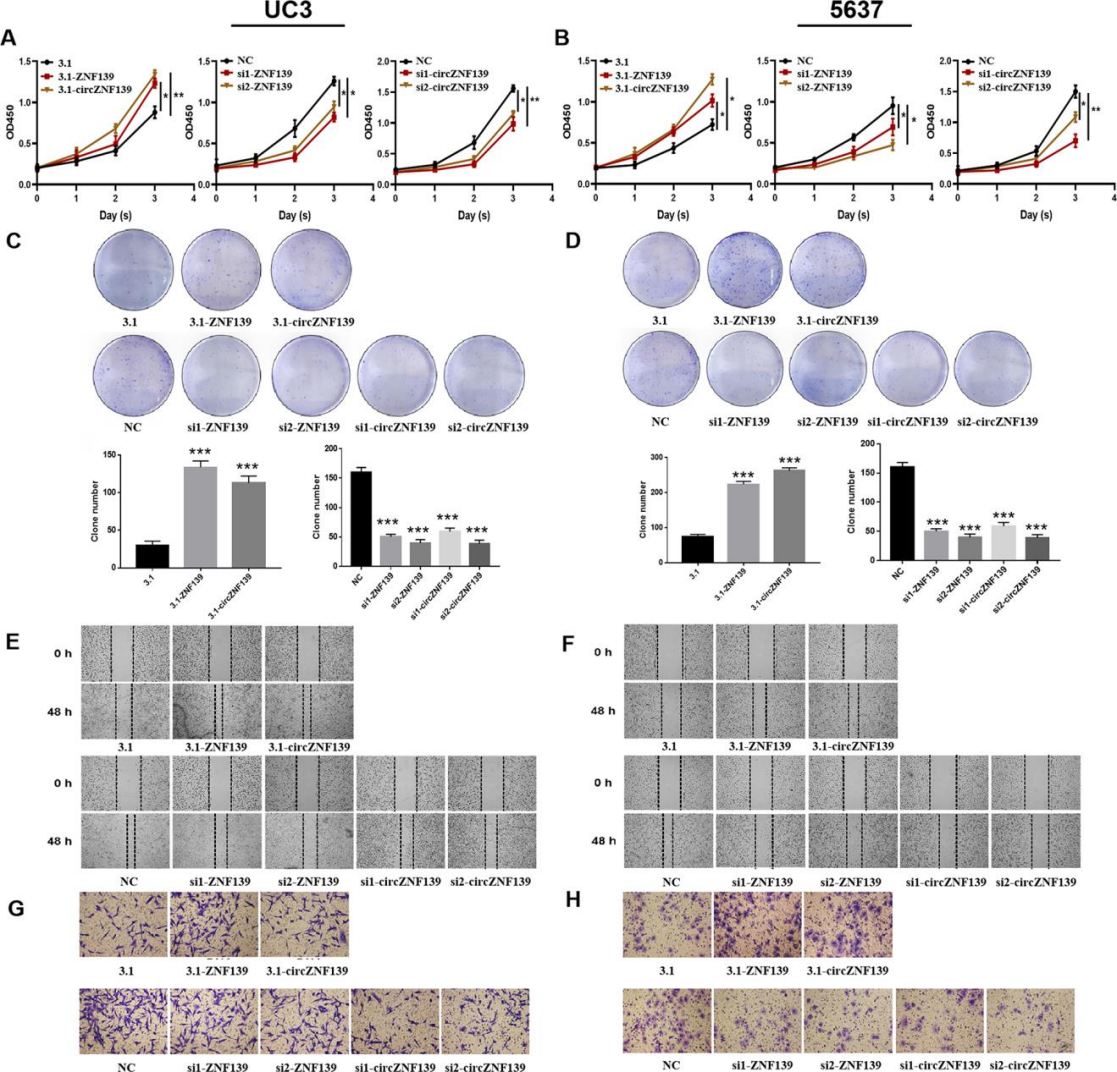
**Cell proliferation, clone, migration, and invasion of UC3 and 5637 cells were evaluated after *ZNF139/circZNF139* overexpression or knockdown.** (**A**–**B**) CCK8 assay was employed to assess the proliferation of UC3 and 5637 cells with *ZNF139/circZNF139* overexpression or knockdown. (**C**–**D**) Crystal violet staining was used to examine the colony formation of UC3 and 5637 cells with *ZNF139/circZNF139* overexpression or knockdown. (**E**–**F**) Scratch wound healing assay was employed to evaluate the migration of UC3 and 5637 cells with *ZNF139/circZNF139* overexpression or knockdown. Images of cell migration at 0 and 48 h transfection are shown at a magnification of 40×. (**G**–**H**) Transwell assay was used to analyze the invasion of UC3 and 5637 cells with *ZNF139/circZNF139* overexpression or knockdown. Images are representative of the cells invading one field at a magnification of 100×. *, *P*<0.05; **, *P*<0.01. ZNF139, zinc finger with KRAB and SCAN domains 1; circ, circular; h, hour.

With an aim to explore the effect of *ZNF139* and *circZNF139* on the biological behavior of BC cells, gain-of- and loss-of-function assays were performed in UC3 and 5637 cells. The 3.1-ZNF139 was constructed and transfected into cells for *ZNF139* overexpression, while two si-ZNF139s (si1-ZNF139 and si2-ZNF139) were transfected into cells for *ZNF139* knockdown. Similarly, 3.1-circZNF139 was for *circZNF139* overexpression, whereas si1-circZNF139 and si2-circZNF139 were for its knockdown. After separate transfection with 3.1-ZNF139, si1-ZNF139, si2-ZNF139, 3.1-circZNF139, si1-circZNF139 or si2-circZNF139 into BC cells, their corresponding efficiencies were determined and confirmed by qRT-PCR assays (Supplementary Figure 2, *P*<0.01, *P*<0.001). The results of CCK-8 assays revealed the proliferative ability of cells in 3.1-ZNF139 or 3.1-circZNF139 groups was evidently enhanced, while that in si-ZNF139s or si-circZNF139s groups was notably attenuated (Figure 6A, 6B, *P*<0.05, *P*<0.01). Turning now to the experimental evidence on clonal assay, the colony formation of BC cells was promoted in UC3 and 5637 cells with *ZNF139* or *circZNF139* overexpression, while that was inhibited in those cells with *ZNF139* or *circZNF139* knockdown (Figure 6C, 6D). In addition, *ZNF139* or *circZNF139* overexpression obviously facilitated cell migration and invasion in UC3 and 5637 cells (Figure 6E–H). Conversely, *ZNF139* or *circZNF139* knockdown evidently suppressed cell migration and invasion in UC3 and 5637 cells (Figure 6E–H). These data collectively indicated that *ZNF139/circZNF139* stimulated the proliferative, clonal, migratory, and invasive potential of BC cells.

### *ZNF139/circZNF139* activates the PI3K/AKT signaling pathway in BC

**Figure 7:**
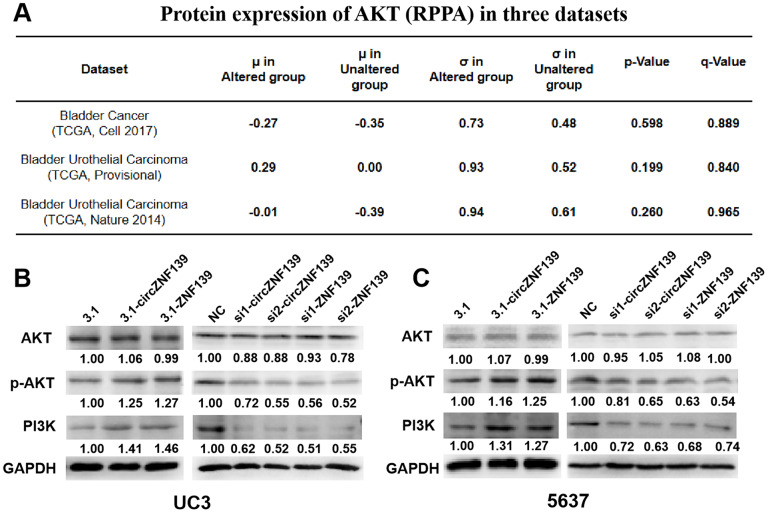
***ZNF139* and its circRNA (*circZNF139*) activates PI3K/AKT signaling pathway in BC cells.** (**A**) The expression of AKT in *ZNF139*-altered and *ZNF139*-unaltered groups in three datasets as analyzed in cBioPortal (http://cbioportal.org) database. (**B**–**C**) The protein levels of AKT, p-AKT, and PI3K in UC3 and 5637 cells treated with 3.1-ZNF139, si1-ZNF139, si2-ZNF139, 3.1-circZNF139, si1-circZNF139, si2-circZNF139 or matched controls (3.1/NC) were determined by western blot assays, respectively. ZNF139/ZKSCAN1, zinc finger with KRAB and SCAN domains 1; circ, circular; si, small interfering; NC, negative control.

Supportive by the aforementioned results, *ZNF139* may function as a transcription factor to affect related signaling pathway, possibly such as PI3K/AKT signaling pathway. According to cBioPortal (http://cbioportal.org) database, it was apparent that the protein expression of AKT was higher in *ZNF139*-altered group than that in *ZNF139*-unaltered group, suggesting that the phosphorylation level of AKT was affected by *ZNF139* expression (Figure 7A). We therefore hypothesized that *ZNF139* and *circZNF139* probably regulated PI3K/AKT signaling pathway in the progression of BC cells. Western blot assays were performed to determine the protein levels of AKT, PI3K and the phosphorylation level of AKT in UC3 and 5637 cells transfected with 3.1-ZNF139, si1-ZNF139, si2-ZNF139, 3.1-circZNF139, si1-circZNF139, si2-circZNF139 or matched controls (3.1/NC). Closer inspection of the results suggested that UC3 cells with 3.1-ZNF139 or 3.1-circZNF139 presented a promotion in the phosphorylation level of AKT and the protein level of PI3K, while cells with si-ZNF139s or si-circZNF139s exerted an opposite effect on their levels (Figure 7B). So was the case of 5637 cells (Figure 7C). By and large, these findings unearthed that *ZNF139* and its circRNA (*circZNF139*) could activate PI3K/AKT signaling pathway in BC cells.

## DISCUSSION

Reportedly, *ZNF139* as a regulatory role in many genes at their transcriptional level is involved in the development and progression of various tumors. Notably, its roles are well studied in gastric cancer, including tumor metastasis, apoptosis, and multidrug resistance [22, 23, 30, 31]. However, no previous study has investigated the relevance and function of *ZNF139* in BC. Through a series of bioinformatics analyses in the present study, we found that ZNF139 was upregulated in BC tissues, including bladder urothelial carcinoma and superficial BC. Meanwhile, its expression was obviously correlated with the disease-free survival of BC patients, but not with their overall survival. These findings possibly indicated that ZNF139 is involved in the occurrence and prognosis of BC.

As a member of the zinc finger protein family, *ZNF139* is a transcription factor [32, 33]. Theoretically, it was located in the nucleus to a great extent. However, according to our bioinformatics analysis, *ZNF139* was mainly located in the nucleus, and secondly common in mitochondrion. *ZNF139* encodes a member of the Kruppel zinc-finger family of proteins with C_2_H_2_ type. According to UniProt, ZNF139 protein contained C_2_H_2_- structure specific to transcription factors. Later mRNA sequencing data sourced from TCGA outlined 7,077 positively genes and 5,942 negatively genes correlated with *ZNF139*. These supported or verified that *ZNF139* could function as a transcription factor with a wide-ranging effect on the transcriptome.

Commonly, circRNAs consist of a large class of non-coding RNAs, generated by a non-canonical back splicing event [11]. As newly discovered molecules among the non-coding RNA network, circRNAs have been identified to be implicated in various cancers [11, 34, 35]. As previously reported, ZNF139 gene could generate two types of RNA, including *ZNF139* mRNA and *circZNF139*, which were both involved in the progression of hepatocellular carcinoma [24]. In our work, the *circZNF139*, a circRNA form of *ZNF139*, was validated by UCSC, DeepBase and circBase databases. This suggested that *circZNF139* (circRNA ID: hsa_circ_0001727) with 668 nt spliced length had the widest expression spectrum, also supportive by the reported uncovers [25, 26]. On the other, qRT-PCR analysis revealed that *ZNF139* and its circRNA (*circZNF139*) were significantly upregulated in BC cell lines. The circular characteristics of *circZNF139* were confirmed by tests of the enzyme RNase R., equal to the results presented in previous study [24].

Numerous studies have concentrated on the role of circRNAs in tumors, in which individual circRNAs exert either suppressive or promoted effects on the progression of tumors [36, 37]. In this study, gain-of and loss-of-function assays collectively unearthed that *ZNF139/circZNF139* had facilitated effects on the proliferative, clonal, migratory, and invasive potential of BC cells. As for its underlying regulatory mechanism, we could obtain some clues from GO and KEGG pathway analyses of co-expressed genes correlated with *ZNF139* in BC. They clearly revealed that one of the significant pathways associated with these genes was PI3K/AKT signaling pathway. In addition, the apparent result of *ZNF139* affecting the phosphorylation level of AKT, obtained from cBioPortal database, further supported the hypothesis that *ZNF139* and its *circZNF139* probably regulated PI3K/AKT signaling pathway in the progression of BC cells. Later western blot assays with overexpression or knockdown of *ZNF139/circZNF139* unveiled that they activated PI3K/AKT signaling pathway in BC cells.

Ultimately, this research contributed to the knowledge regarding the relevance and function of *ZNF139/circZNF139* in BC. The main finding emerging from this study is the possible regulatory mechanism in which *ZNF139/circZNF139* promotes cell proliferation, migration and invasion via the activation of PI3K/AKT signaling pathway in BC. These findings, despite preliminary, first disclosed the synergistic effect of *ZNF139/circZNF139* on BC. Undoubtedly, the few limitations characterizing this study cannot be ignored. First, related verification was only conducted in cell lines, not including animal models or patients’ samples. Second, the underlying mechanism about how *circZNF139* regulates PI3K/AKT signaling pathway in BC is unexplored. Third, the relevance and function of *circZNF139* in other tumors is absent. Further studies, which take these limitations into account, will need to be undertaken.

## CONCLUSIONS

To sum up, this work reveals one possible regulatory mechanism in which *ZNF139* and *circZNF139* cooperate closely with each other to promote cell proliferation, migration and invasion via the activation of PI3K/AKT signaling pathway in BC. This provides a theoretical basis for BC pathogenesis, and potential therapeutic markers for BC treatment.

## MATERIALS AND METHODS

### CRN and Oncomine analyses

CRN, freely available at http://syslab4.nchu.edu.tw/CRN, is known as the first public database which provides phenotype-specific coding-transcript/long non-coding RNA expression profiles and mRNA–long non-coding RNA co-expressed networks in cancer cells [38]. It consists of cancer RNA-Seq data sets in the TCGA, SRA and GEO databases. In this work, this open source was employed to evaluate the transcript expressions of ZNF139 in bladder urothelial carcinoma at varied stages (Stage I, II, III, IV) relative to adjacent normal. As the world’s largest oncogene chip database and integrated data mining platform, Oncomine (http://www.oncomine.org) contains 715 datasets and 86,733 pairs of cancer and normal tissues [39]. The Oncomine Platform—from web applications to translational bioinformatics services—has laid a foundation for ground-breaking discoveries with scalability, high quality, consistency and standardized analysis. *ZNF139* expression was assessed in Dyrskjot Bladder 3 and Sanchez-Carbayo Bladder 2 tissues, and associated *P*-value < 0.01 was considered significant.

### GeneCards and UniProt analyses

Featured in a searchable, integrative database, GeneCards provides comprehensive, user-friendly information concerning all annotated and predicted human genes, which is accessed via the website https://www.genecards.org/. It automatically integrates gene-centric data from more than 80 digital sources, encompassing the following information—genome, transcriptome, proteome, genome, clinic and so forth [40]. Here, GeneCards was used to explore the subcellular localization for *ZNF139*. Characterized by entirety, high quality and free access, UniProt provides the scientific community with resource of protein sequence and functional information, which can be accessed at http://www.uniprot.org/ [41]. This database was therefore employed for structural analysis of *ZNF139*.

### LinkedOmics analysis

Available at http://www.linkedomics.org/login.php, the LinkedOmics is well acknowledged as a publicly available portal for analyzing multi-omics data from all 32 TCGA Cancer types [42]. This web platform contains three analytical modules- *LinkFinder*, *LinkInterpreter* and *LinkCompare*. Among them, on the one hand, the *LinkFinder* module of LinkedOmics was employed for analyzing genes differentially expressed in correlation with *ZNF139* in the TCGA BLCA cohort (n=408). Pearson’s correlation coefficient was used for statistical analysis. All results were visualized as volcano plots and heat maps. On the other hand, the *LinkInterpreter* module of LinkedOmics performs pathway and network analyses of differentially expressed genes to derive biological insights from the association results. Data obtained from the *LinkFinder* results were signed and ranked according to the following criterion: Rank Criteria: *p*-value; Minimum Number of Genes:3; Simulations:1000. GSEA was applied for analyses of GO and KEGG pathways about the genes co-expressed with *ZNF139*.

### cBioPortal analysis

The cBio Cancer Genomics Portal (http://cbioportal.org) offers an open-access resource with more than 5,000 tumor samples for interactively exploring genomics data sets of multidimensional cancer [43]. c-BioPortal was employed to analyze the association of *ZNF139* expression with the overall survival and disease-free survival. Moreover, the expressions of AKT1, AKT2, AKT3 in ZNF139-altered and ZNF139-unaltered groups were analyzed in this database.

### UCSC analysis

UCSC Genome Browser (http://genome.ucsc.edu/), a web-based and open source platform, efficiently provides the speedy, scalable display of sequence alignments and annotations landscaped against a good deal of genome assemblies under quality reference [44]. This database was utilized to evaluate the circRNA-formed ability of *ZNF139*.

### DeepBase and circBase analyses

Freely accessible at https://omictools.com/deepbase-tool, deepBase is a platform designed for annotating and discovering small RNAs, long non-coding RNAs as well as circRNAs according to sequencing data of next generation [45]. Here, the expression level of several circRNAs formed by *ZNF139* in various tumor cells was predicted in this database. Moreover, the expression level of several circRNAs formed by ZNF139 in various tumor samples was further evaluated in circBase database, which provides scripts to identify known and novel circRNAs in sequencing data with free access at http://www.circbase.org/ [46].

### Cell culture and transfection

The human normal SV-HUC-1 cell line and five BC cell lines (UC3, 5637, T24, EJ and J82) were purchased from Cell Bank of Type Culture Collection of Chinese Academy of Sciences (Shanghai, China). They were maintained in Dulbecco’s modified Eagle’s medium (Thermo Fisher Scientific, Waltham, MA, USA) with 10% (v/v) fetal bovine serum (Invitrogen, Carlsbad, CA, USA) in a humidified atmosphere of 5% (v/v) CO_2_ at 37°C.

The pcDNA 3.1, 3.1-ZNF139 and 3.1-circZNF139 were separately constructed by Sangon Biotech (Shanghai, China). Small interfering (si) RNAs against *ZNF139* (si1-ZNF139 and si2-ZNF139), *circZNF139* (si1-circZNF139 and si2-circZNF139) and their corresponding NCs were also synthesized by SangonBiotech (Shanghai, China). The sequences used in this work were listed as follows: si1-ZNF139: 5’-GCACUAUUCACAGCGGAUU-3’ (sense); si2-ZNF139: 5’-GCCACAGUUCCAAUCUCAU-3’ (sense); si1-circZNF139: 5’-AGUGCUCCUUAUCAUUUCU-3’ (sense); si2-circZNF139: 5’-GUCAGUGCUCCUUAUCAUU-3’ (sense). The UC3 and 5637 cell lines were plated in 6-well platesfor18 h, followed by being transfected with indicated siRNAs or plasmids using Lipofectamine 2000 (Invitrogen) in line with the manufacturer’s instructions. Approximately 48 h post transfection, cells were collected for the following assays.

### RNA isolation and qRT-PCR assay

In accordance with the manufacturer’s instructions, total RNA was extracted using TRIzol reagent (Invitrogen, Carlsbad, CA) and then reversely transcribed to a single-stranded cDNA using Reverse Transcription SystemKit (Takara, Dalian, China). qRT-PCR assays were performed by using SYBR Premix Ex Taq (Takara Biotech, Japan) on an ABI 7900 system (Applied Biosystems, Foster City, CA, USA). Relative gene expression was calculated using the 2^-ΔΔCt^ method. The housekeeping gene glyceraldehyde 3-phosphate dehydrogenase (GAPDH) was used as the internal control. The PCR primers were listed as follows: *ZNF139*, forward 5’-TGTAATGAGTGCGGGAAGG-3’ and reverse 5’-AATCAGGTATGAGTTTCGGTTG-3’; *circZNF139*, forward 5’-AGTCCCACTTCAAACATTCGTCT-3’ and reverse 5’-CACCTTCACTATTACGATACCATCC-3’; *GAPDH*, forward 5’-ACGGATTTGGTCGTATTGGGCG-3’ and reverse 5’-GCTCCTGGAAGATGGTGATGGG-3’.

### RNase R treatment

The RNase R treatment was performed in reference to the previous report [24]. In brief, 5 μg of total RNA was incubated for 15 min at 37°C in the presence or absence of RNase R (Epicentre Biotechnologies, Madison, WI, USA) at a concentration of 3 U·μg^-1^. They were separately labeled as RNase R^+^ group and RNase R^-^ group. After purified by phenol–chloroform extraction, the RNA was treated for qRT-PCR assay.

### Cell proliferation, colony formation, migration, and invasion assays

Cell Counting Kit-8 (CCK-8) assays were performed to evaluate the proliferative ability of UC3 and 5637 cells. Cells were cultured in 96-well plates and incubated for 24, 48 and 72 hours (h). The CCK-8 solution (Beyotime Institute of Biotechnology, Shanghai, China) was added to measure the optical density. The absorbance values at each point were measured at 450 nm. At least triplicate samples were evaluated for each treatment.

For colony formation assays, UC3 and 5637 cells (5 × 10^2^ cells per well) were seeded in a 6-well plate and cultured for 14 days after treatment. Colonies were fixed with 10% formaldehyde for 5 min, and stained with 1% crystal violet for 20 min and counted.

The scratch wound-healing assays were conducted to evaluate the migratory ability of UC3 and 5637 cells. The cells were plated on 6-well plates and scraped by a pipette tip to produce uniform wounds prior to transfection. The exfoliated cells were removed by washing each well thrice with PBS. The initial distance (0 h) and the distances after 48 h of scratching were microscopically detected. The images were presented at a magnification of 40× for each group. At least three biological replicates were conducted.

For the invasion assay, the transwell system and Matrigel BD Biosciences (New York, NY, USA) were employed in reference to the manufacturers’ protocols. The upper chamber was treated by Matrigel at 37°C for 2 h and subsequently, transfected cells suspended in serum-free medium were added in. After incubation for 48 h, the cells remaining in the upper chamber were removed by cotton tip carefully, and the cells at the bottom of the insert were fixed, stained with 1% crystal violet, and counted under a microscope. The images of stained cells (invaded cells) in random fields were shown at a magnification of 100× for each group. At least three biological replicates were conducted.

### Western blot assay

Western blot assay was performed using the standard procedure as reported [47]. Briefly, cells were lysed by cell lysis buffer (Beyotime Biotechnology Shanghai, China). Afterwards, the collected protein samples were separated by SDS-PAGE. After blocking, the membrane was incubated with primary antibodies—AKT (1:1000, Catalog: A10605, ABclonal Technology); p-AKT-s473 (1:1000, Catalog: AP0140, ABclonal Technology); PI3K (1:1000, Catalog: A11177, ABclonal Technology); GAPDH (1:1000, Catalog: AC002, ABclonal Technology). The incubation was next followed with the horseradish peroxidase-conjugated goat anti-mouse (1:10000, Catalog: AS003, ABclonal Technology) or anti-rabbit (1:10000, Catalog: AS014, ABclonal Technology). GAPDH was used as internal control.

### Statistics

Data in this study are presented as the mean ± SD. A Student’s *t*-test was used to analyze the statistical significance among groups using GraphPad Prism 8.0 (La Jolla, CA, USA). Value of *P*< 0.05 was considered to be statistically significant. All experiments were independently conducted at least three times.

## Supplementary Material

Supplementary Figures

Supplementary Tables
