# Correction for: *ZNF139*/*circZNF139* promotes cell proliferation, migration and invasion via activation of PI3K/AKT pathway in bladder cancer

**DOI:** 10.18632/aging.204132

**Published:** 2022-06-14

**Authors:** Jie Yao, Kaiyu Qian, Chen Chen, Xiaoping Liu, Donghu Yu, Xin Yan, Tongzu Liu, Sheng Li

**Affiliations:** 1 Department of Biological Repositories, Zhongnan Hospital of Wuhan University, Wuhan, 430071, China; 2 Human Genetics Resource Preservation Center of Hubei Province, Wuhan, 430071, China; 3 Department of Urology, Zhongnan Hospital of Wuhan University, Wuhan, 430071, China

Original article: Aging. 2020; 12:9915–9934.  . https://doi.org/10.18632/aging.103256

**This article has been corrected**: The authors noticed errors in **Figure 6**. As a result of misfiling the data, the images in panel 6C showing UC3 cells transfected with si1- and si2-circZNF139 were the same as the images in panel 6D showing 5637 cells transfected with si1- and si2-circZNF139. The authors corrected panels 6C and 6D in **Figure 6** by using representative images from the original sets of experiments. Similarly, the images of cells obtained 48 h after si1-- and si2-circZNF139 transfection were mixed up during preparation of panel 6F and replaced. The images showing invasion by UC3 cells transfected with si1- and si2-ZNF139 were replaced in panel 6G, as was the image of 5637 cells transfected with 3.1 in panel 6H. All images were derived from the initial sets of experiments. The authors stated that these alterations do not affect the results or conclusions of this work and apologized for any inconvenience caused.

New **Figure 6** is presented below.

**Figure 6:**
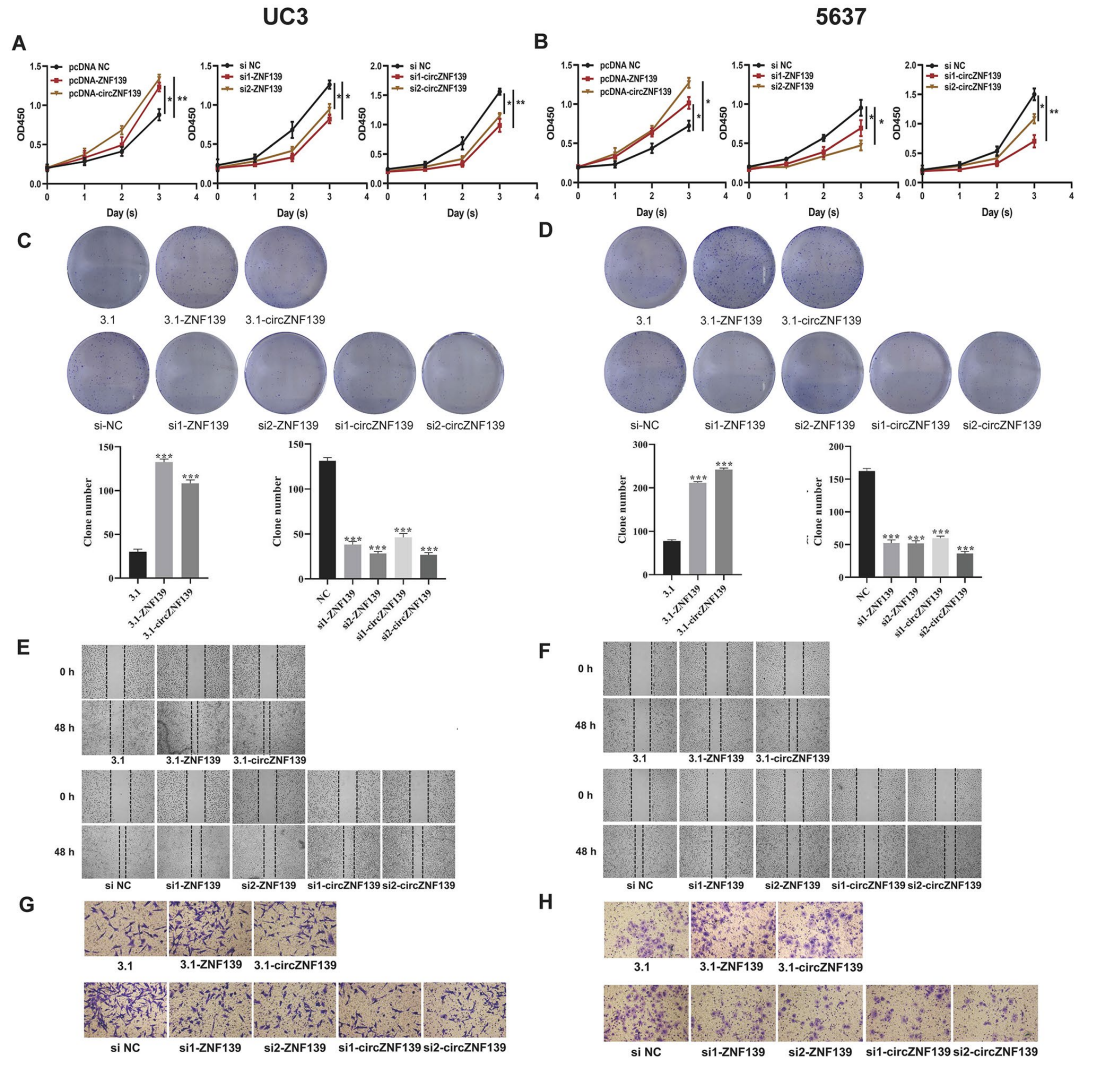
**Cell proliferation, clone, migration, and invasion of UC3 and 5637 cells were evaluated after *ZNF139/circZNF139* overexpression or knockdown.** **(A–B) **CCK8 assay was employed to assess the proliferation of UC3 and 5637 cells with *ZNF139/circZNF139* overexpression or knockdown. (**C–D**) Crystal violet staining was used to examine the colony formation of UC3 and 5637 cells with *ZNF139/circZNF139* overexpression or knockdown. (**E–F**) Scratch wound healing assay was employed to evaluate the migration of UC3 and 5637 cells with *ZNF139/circZNF139* overexpression or knockdown. Images of cell migration at 0 and 48 h transfection are shown at a magnification of 40×. (**G–H**) Transwell assay was used to analyze the invasion of UC3 and 5637 cells with *ZNF139/circZNF139* overexpression or knockdown. Images are representative of the cells invading one field at a magnification of 100×. *, *P*<0.05; **, *P*<0.01. ZNF139, zinc finger with KRAB and SCAN domains 1; circ, circular; h, hour.

