# Supplementary material for: ZNF139/circZNF139 promotes cell proliferation, migration and invasion via activation of PI3K/AKT pathway in bladder cancer
Source: Aging (Albany NY). 2020 May 26;12(10):9915–34. doi: 10.18632/aging.103256 (PMC7288921; doi:10.18632/aging.103256)
Supplement: Supplementary Figures [file aging-12-103256-s002..pdf]

SUPPLEMENTARY FIGURES

organism	position (genome browser link)	strand	circRNA ID	genomic length	spliced length	samples	scores	repeats	annotation	best transcript	gene symbol	circRNA study
hsa	chr7:99621041-99621930	+	has_circ_0001727	889	668	Hs68_control, Hs68_Rnase, adipose, EPC, HUAEC, LCL, platelets, WAS2, cd_19, cd_34, HEK293, cerebellum, diencephalon, frontal_cortex, occipital_lobe, parietal_lobe, Sy5y_exp1_D0, Sy5y_exp1_D2, Sy5y_exp1_D4, SY5Y_exp2_D0, SY5Y_exp2_D4, SY5Y_exp2_D8, temporal_lobe, A549, Ag04450, Bj, Gm12878, H1hesc, Helas3, Hepg2, Hsmm, Huvec, K562, Mcf7, Nhek, Nhlf, Sknshra	61, 1247, 4, 4, 2, 5, 5, 7, 19, 11, 2, 260, 425, 2029, 1427, 570, 37, 22, 18, 24, 9, 7, 911, 148, 134, 114, 155, 150, 112, 119, 15, 39, 198, 47, 243, 3, 198	None	ANNOTATED, CDS, coding, INTERNAL, OVCODE, OVEXON, UTR5	NM_003439	ZKSCAN1	Jeck2013, Maass2017, Memczak2013, Rybak2015, Salzman2013
mmu	chr5: 138534147-138535367	+	mmu_circ_0012225	1220	661	forebrain, olfactory_bulb	17, 2	NA	ANNOTATED, CDS, coding, INTERNAL, OVCODE, OVEXON, UTR5	ENSMUST00000019660	Zkscan1	Rybak2015
hsa	chr7: 99621041-99627998	+	has_circ_0135233	6957	887	frontal_cortex	9	NA	ANNOTATED, CDS, coding, INTERNAL, OVCODE, OVEXON, UTR5	ENST00000324306.6	ZKSCAN1	Rybak2015
hsa	chr7: 99633596-99633805	-	has_circ_0135235	209	2019	temporal_lobe	7	NA	ANTISENSE, Coding, INTERNAl OVEXON, UTR3	ENST00000324306.6	ZKSCAN1	Rybak2015

Supplementary Figure 1. The expression of circRNAs formed by *ZNF139* in various samples in accordance with the search of *ZNF139* in circBase (http://www.circbase.org/) database. The red box indicates the circZNF139 (circRNA ID: hsa_circ_0001727) with 668 nt spliced length who has the widest expression spectrum. ZNF139, zinc finger with KRAB and SCAN domains 1; circ, circular.

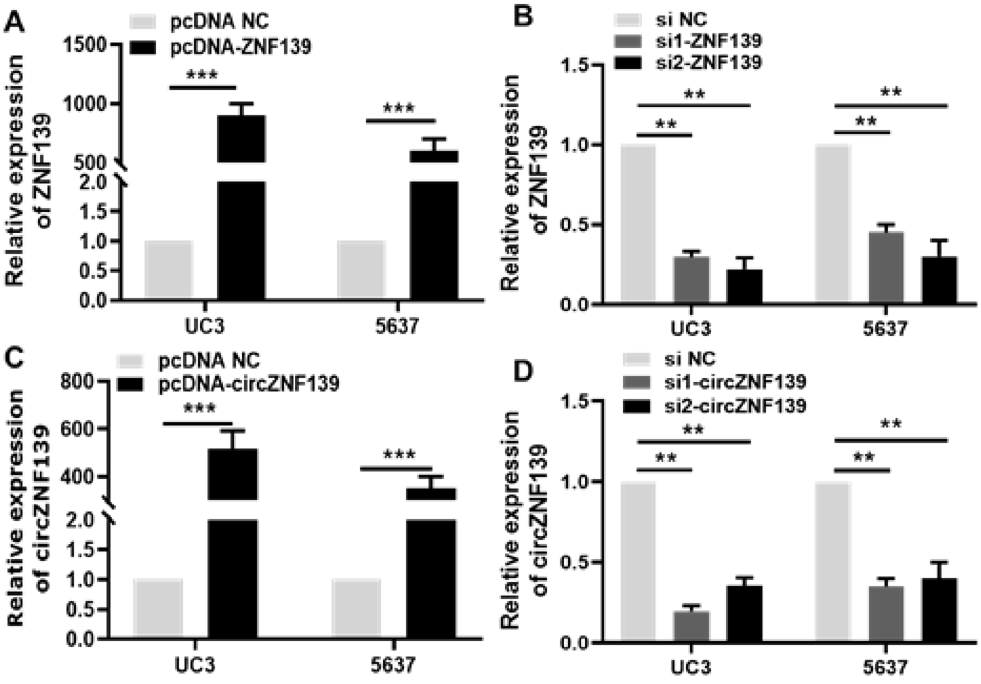

Supplementary Figure 2. The overexpression and knockdown efficiencies of *ZNF139/circZNF139* were detected in UC3 and 5637 cells by qRT-PCR assay. (**A**–**B**) The expression of ZNF139 was measured in UC3 and 5637 cells after respectively transfected with pcDNA-ZNF139/pcDNA NC, or si1-ZNF139/si2-ZNF139/si NC. (**C**–**D**) The expression of circZNF139 was measured in UC3 and 5637 cells after respectively transfected with pcDNA-circZNF139/pcDNA NC, or si1-circZNF139/si2-circZNF139/si NC. **, *P*<0.01; ***, *P*<0.001. circ, circular; ZNF139, zinc finger with KRAB and SCAN domains 1; qRT-PCR, quantitative real-time polymerase chain reaction; NC, negative control; si, small interfering.
