# Supplementary material for: ZNF139/circZNF139 promotes cell proliferation, migration and invasion via activation of PI3K/AKT pathway in bladder cancer
Source: Aging (Albany NY). 2020 May 26;12(10):9915–34. doi: 10.18632/aging.103256 (PMC7288921; doi:10.18632/aging.103256)
Supplement: Supplementary Tables [file aging-12-103256-s001..pdf]

SUPPLEMENTARY TABLES

Supplementary Table 1. Top-50 significant genes positively correlated with *ZKSCAN1* (*i.e. ZNF139*) in BC.

NO.	Query	Statistic	*P*-value	FDR (BH)	Event SD	Event TD
1	ZKSCAN1	1	1.00E-87	1.00E-83	408	408
2	MLL5	0.788332	1.13E-87	1.14E-83	408	408
3	AKAP9	0.778419	4.03E-84	2.69E-80	408	408
4	LMTK2	0.774001	1.35E-82	6.75E-79	408	408
5	MYSM1	0.772319	5.01E-82	2.01E-78	408	408
6	ZNF678	0.764579	1.85E-79	6.16E-76	408	408
7	LOC100190986	0.750676	4.32E-75	1.24E-71	408	408
8	MLL2	0.750307	5.59E-75	1.40E-71	408	408
9	ZNF81	0.736745	5.40E-71	1.20E-67	408	408
10	CHD6	0.734598	2.19E-70	4.39E-67	408	408
11	KRIT1	0.729158	7.17E-69	1.31E-65	408	408
12	MLL3	0.727939	1.55E-68	2.59E-65	408	408
13	UBN2	0.725239	8.40E-68	1.30E-64	408	408
14	LCOR	0.724151	1.65E-67	2.36E-64	408	408
15	TRRAP	0.718551	5.08E-66	6.79E-63	408	408
16	SMG1	0.717804	7.98E-66	9.99E-63	408	408
17	ZNF827	0.717087	1.23E-65	1.45E-62	408	408
18	SHPRH	0.716103	2.21E-65	2.47E-62	408	408
19	C12orf51	0.712749	1.62E-64	1.70E-61	408	408
20	ZNF192	0.712677	1.69E-64	1.70E-61	408	408
21	EIF2C4	0.711538	3.31E-64	3.16E-61	408	408
22	FLJ10213	0.709187	1.30E-63	1.19E-60	408	400
23	KIAA1109	0.70631	6.86E-63	5.98E-60	408	408
24	EP300	0.70549	1.10E-62	9.17E-60	408	408
25	C20orf94	0.703129	4.21E-62	3.37E-59	408	407
26	HEATR5B	0.701856	8.63E-62	6.60E-59	408	408
27	DPY19L3	0.701802	8.89E-62	6.60E-59	408	408
28	ZNF791	0.701417	1.10E-61	7.83E-59	408	408
29	LOC90834	0.701371	1.13E-61	7.83E-59	408	404
30	NR2C2	0.701226	1.23E-61	8.11E-59	408	408
31	CCNT2	0.70119	1.25E-61	8.11E-59	408	408
32	ARID2	0.695761	2.56E-60	1.60E-57	408	408
33	LOC646471	0.689137	9.23E-59	5.61E-56	408	408
34	ZNF518A	0.688392	1.37E-58	8.10E-56	408	408
35	CCNT1	0.687117	2.71E-58	1.55E-55	408	408
36	ADNP	0.687013	2.86E-58	1.59E-55	408	408
37	LOC100271836	0.685722	5.65E-58	3.06E-55	408	408
38	GPATCH8	0.684815	9.11E-58	4.81E-55	408	408
39	NCOA2	0.684468	1.09E-57	5.62E-55	408	408
40	ANKIB1	0.684213	1.25E-57	6.26E-55	408	408
41	TAF1	0.684072	1.34E-57	6.57E-55	408	408

42	RALGAPA2	0.68289	2.49E-57	1.19E-54	408	408
43	KIAA1267	0.681432	5.32E-57	2.48E-54	408	408
44	REST	0.681275	5.77E-57	2.63E-54	408	408
45	CHD2	0.679689	1.31E-56	5.83E-54	408	408
46	KLHL11	0.679388	1.53E-56	6.65E-54	408	407
47	SRRM2	0.678389	2.55E-56	1.09E-53	408	408
48	BTAF1	0.677899	3.27E-56	1.37E-53	408	408
49	ATAD2B	0.676497	6.68E-56	2.73E-53	408	408
50	ZNF292	0.675971	8.73E-56	3.50E-53	408	408

ZNF139/ZKSCAN1, zinc finger with KRAB and SCAN domains 1; BC, bladder cancer.

Supplementary Table 2. Top-50 significant genes negatively correlated with *ZKSCAN1* (*i.e. ZNF139*) in BC.

NO.	Query	Statistic	*P*-value	FDR (BH)	Event SD	Event TD
1	AP2S1	-0.65333	5.13E-51	1.45E-48	408	408
2	SNRPC	-0.61244	2.29E-43	2.91E-41	408	408
3	NCRNA00152	-0.61084	4.35E-43	5.22E-41	408	408
4	SEC61B	-0.60979	6.59E-43	7.72E-41	408	408
5	PSMB7	-0.60599	2.94E-42	3.31E-40	408	408
6	PFN1	-0.60308	9.06E-42	9.82E-40	408	408
7	PSMC3	-0.60046	2.48E-41	2.58E-39	408	408
8	C16orf42	-0.59934	3.81E-41	3.94E-39	408	408
9	ATOX1	-0.59884	4.61E-41	4.74E-39	408	408
10	PSMD9	-0.59757	7.48E-41	7.42E-39	408	408
11	POMP	-0.595	1.97E-40	1.89E-38	408	408
12	RDBP	-0.59466	2.24E-40	2.12E-38	408	408
13	C1orf212	-0.59384	3.05E-40	2.83E-38	408	408
14	NAA10	-0.58779	2.86E-39	2.42E-37	408	408
15	GUK1	-0.5872	3.55E-39	2.98E-37	408	408
16	LOC541471	-0.58705	3.75E-39	3.13E-37	408	408
17	BRMS1	-0.58684	4.05E-39	3.36E-37	408	408
18	CCDC85B	-0.58508	7.68E-39	6.21E-37	408	408
19	EDF1	-0.58428	1.02E-38	8.22E-37	408	408
20	DRAP1	-0.58403	1.12E-38	8.98E-37	408	408
21	PSMB3	-0.5813	3.00E-38	2.28E-36	408	408
22	PSMD13	-0.58042	4.11E-38	3.09E-36	408	408
23	MRPL14	-0.57993	4.89E-38	3.62E-36	408	408
24	MYL6	-0.57916	6.45E-38	4.74E-36	408	408
25	POLR2L	-0.57579	2.12E-37	1.47E-35	408	408
26	SNF8	-0.57375	4.33E-37	2.96E-35	408	408
27	CLIC1	-0.57339	4.91E-37	3.33E-35	408	408
28	PSMB6	-0.57339	4.92E-37	3.33E-35	408	408
29	DPM2	-0.57085	1.18E-36	7.81E-35	408	408
30	RNASEK	-0.57047	1.35E-36	8.88E-35	408	408
31	ORAI1	-0.57039	1.39E-36	9.10E-35	408	408
32	SSSCA1	-0.56773	3.47E-36	2.21E-34	408	408
33	NUDC	-0.56509	8.50E-36	5.26E-34	408	408
34	RHOG	-0.56358	1.41E-35	8.64E-34	408	408
35	C12orf44	-0.56282	1.83E-35	1.11E-33	408	408
36	MED27	-0.55808	8.90E-35	5.08E-33	408	408
37	MRPS24	-0.55754	1.06E-34	5.99E-33	408	408
38	YIF1A	-0.55694	1.29E-34	7.19E-33	408	408
39	METTL11A	-0.55583	1.87E-34	1.02E-32	408	408
40	FAM176B	-0.55518	2.31E-34	1.26E-32	408	408
41	PSMC1	-0.55472	2.68E-34	1.45E-32	408	408
42	AURKAIP1	-0.55419	3.19E-34	1.71E-32	408	408
43	ARL2	-0.55193	6.67E-34	3.52E-32	408	408

44	PSMB1	-0.55185	6.83E-34	3.59E-32	408	408
45	PTRH1	-0.55159	7.44E-34	3.88E-32	408	408
46	CSNK2B	-0.55129	8.18E-34	4.25E-32	408	408
47	RHOC	-0.54884	1.80E-33	9.21E-32	408	408
48	C9orf89	-0.54758	2.69E-33	1.36E-31	408	408
49	CLTA	-0.54696	3.28E-33	1.63E-31	408	408
50	MRPL17	-0.54665	3.63E-33	1.79E-31	408	408

ZNF139/ZKSCAN1, zinc finger with KRAB and SCAN domains 1; BC, bladder cancer.
